# Calcification, Storm Damage and Population Resilience of Tabular Corals under Climate Change

**DOI:** 10.1371/journal.pone.0046637

**Published:** 2012-10-04

**Authors:** Joshua S. Madin, Terry P. Hughes, Sean R. Connolly

**Affiliations:** 1 Department of Biological Sciences, Macquarie University, Sydney, Australia; 2 ARC Centre of Excellence for Coral Reef Studies, James Cook University, Townsville, Australia; 3 School of Marine Biology and Tropical Biology, James Cook University, Townsville, Australia; University of California Merced, United States of America

## Abstract

Two facets of climate change–increased tropical storm intensity and ocean acidification–are expected to detrimentally affect reef-building organisms by increasing their mortality rates and decreasing their calcification rates. Our current understanding of these effects is largely based on individual organisms’ short-term responses to experimental manipulations. However, predicting the ecologically-relevant effects of climate change requires understanding the long-term demographic implications of these organism-level responses. In this study, we investigate how storm intensity and calcification rate interact to affect population dynamics of the table coral *Acropora hyacinthus*, a dominant and geographically widespread ecosystem engineer on wave-exposed Indo-Pacific reefs. We develop a mechanistic framework based on the responses of individual-level demographic rates to changes in the physical and chemical environment, using a size-structured population model that enables us to rigorously incorporate uncertainty. We find that table coral populations are vulnerable to future collapse, placing in jeopardy many other reef organisms that are dependent upon them for shelter and food. Resistance to collapse is largely insensitive to predicted changes in storm intensity, but is highly dependent on the extent to which calcification influences both the mechanical properties of reef substrate and the colony-level trade-off between growth rate and skeletal strength. This study provides the first rigorous quantitative accounting of the demographic implications of the effects of ocean acidification and changes in storm intensity, and provides a template for further studies of climate-induced shifts in ecosystems, including coral reefs.

## Introduction

Widespread changes in marine ecosystem function, species abundances and geographic ranges are all a likely consequence of environmental trends associated with ongoing anthropogenic effects on ocean pH [1], sea temperatures [2], and storm intensity [3]. On coral reefs, our understanding of the future ecological effects of these environmental changes is based overwhelmingly on extrapolation from short-term experimental studies of individual organisms’ physiological and biomechanical responses [4], [5]. However, predicting the large-scale, long-term effects of climate change requires a better understanding of how climate change will alter demography and population dynamics over decadal time-spans [6], [7]. A consensus is emerging that such predictions require the coupling of population-dynamic models and environmental variables via biophysical (mechanistic) models [8], [9], [10], because such models are built on principles of physical relationships that will be unchanged in future environments. Moreover, a stage- or size-structure framework is required, because homogeneous population measures, such as abundance or percent cover, do not capture important changes in demographic rates as individuals grow [11], [12] and because various life history stages are likely to respond differently to changes in environmental conditions [13], [14].

On coral reefs, mortality rates of coral colonies are elevated by severe summer storms and cyclones, which dislodge colonies from the substrate, particularly in wave-exposed, highly productive habitats like reef crests [12], [15]. Such mechanical disturbances limit the dominance of fast-growing, mechanically unstable coral growth forms, and facilitates the maintenance of high local diversity in coral assemblages [16]. Predicted increases in the intensity of storms and cyclones [3] are likely to increase mortality in biomechanically vulnerable species. Moreover, the effects of storms may be exacerbated by declining rates of calcification caused by ocean acidification and thermal stress [17]. Decreased calcification by corals, if manifested as slower colony growth rates [18], may also have implications for lifetime reproductive output, which is strongly related to colony size and longevity [19].

**Figure 1:**
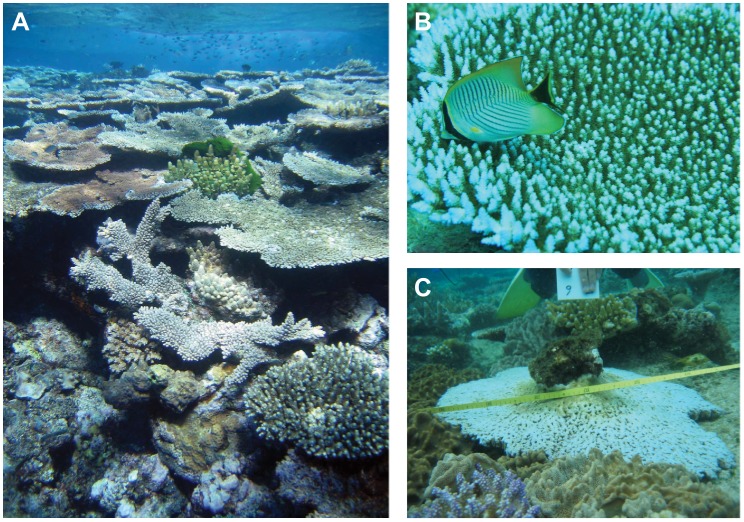
Tabular corals that provide habitat structure, shelter and food for associated reef organisms. (A) Wave-exposed coral communities are often dominated by tabular corals (photo: Andrew Baird). (B) The obligate corallivore, *Chaetodon trifascialis*, feeds almost exclusively on the pandemic study species, *Acropora hyacinthus* (photo: Morgan Pratchett). (C) Tabular growth forms are particularly vulnerable to mechanical dislodgement during summer storms.

Coral species with tabular growth forms are particularly important ecosystem engineers on wave-exposed Indo-Pacific reefs (Figure 1A). They grow and calcify rapidly compared to other growth forms, allowing them to dominate reef crest communities [20], [21]. Consequently, they are major contributors to calcification and reef accretion in these habitats. Table corals are key contributors to reef structural complexity. They harbour distinctive understory communities [21] and provide shelter from predation and high flow for many mobile species, especially small fishes [22] and juvenile parrotfishes, which as adults play a crucial functional role controlling macroalgae on reefs [23]. Some tabular species are essential prey of corallivorous butterflyfishes, which decline markedly in abundance without them [24] (Figure 1B). However, the same traits that make table corals important ecosystem engineers also make them particularly susceptible to climate change. They are vulnerable to dislodgement by storm-generated waves (Figure 1C) [12], [16] and susceptible to ocean acidification, thermal stress, coral bleaching and disease [25], [26].

In this study, we examine the effects of changes in storm intensity and calcification rates on population growth of the ecologically dominant table coral *Acropora hyacinthus*, a pandemic reef-building species on wave-exposed Indo-Pacific reefs [27]. We compare estimates of coral population growth under atmospheric CO_2_ scenarios for the Pre-Industrial Revolution (PIR), present day, and projections under two future climate scenarios. We incorporate into this model: (1) shifts in aragonite saturation state (Ω_arag_) driven by increasing pCO_2_ and sea surface temperature (SST) [28] based on a range of empirically-calibrated relationships between Ω_arag_ and SST and coral calcification rate [5], [29], [30], and (2) a field-validated mechanistic model relating the severity of tropical storm events to the dislodgment mortality of corals of different sizes [12]. Reductions in calcification may be manifested in a combination of two ways: by reduced colony growth rates, and by reduced skeletal density [17], [4]. A decline in colony growth reduces survival and reproductive rate, since colonies will be in smaller, less fecund, and remain in more vulnerable size classes for longer than under normal growth rates [31]. Conversely, reduced skeletal density increases the vulnerability of larger colonies to dislodgment during storms. We therefore consider both types of responses to calcification, to capture the range within which the true response will lie. Because ocean acidification is expected to decrease the mechanical integrity of reef substrate due to declines in inorganic cementation and increases in bioerosion [32], and this, in turn, increases the risk of colony dislodgment during storms [33], we also consider the potential effects of decreases in the strength of reef substrate. Therefore, we examine 12 scenarios in all, corresponding to combinations of (i) weak, intermediate, or strong relationships between calcification and Ω_arag_ and SST, (ii) whether growth rate and/or skeletal density decline as calcification decreases, and (iii) whether reef substrate strength is weak or strong, reflecting both contemporary spatial variation in reef lithification [34], and the potential for a future weakening of substrate strength due to ocean acidification.

## Materials and Methods

### Reef Mechanical Environment

The hydrodynamic dislodgement of coral colonies can be expressed as the dimensionless inequality [12]:
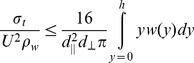
(1)


**Figure 2:**
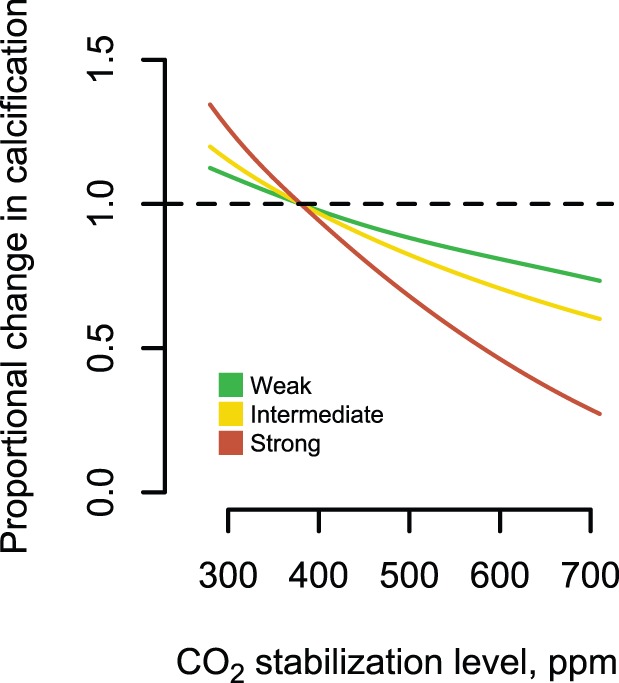
Predicted proportional changes in calcification rate as a function of stabilized atmospheric CO_2_ scenarios. Changes are shown relative to present-day (380 ppm) for three published calcification responses: weak [30], intermediate [29] and strong [5].

The left-hand side of the equation describes the mechanical environment, where *σ_t_* is the limiting material tensile strength (either reef substrate or coral skeleton; Nm^−2^), *U* is the expected yearly maximum water velocity (ms^−1^), and *ρ_w_* is seawater density (∼1025 kgm^−3^). The right-hand side–the Colony Shape Factor (*CSF*)–is a measure of mechanical vulnerability described by the projection of colony shape above the substrate (*w*(*y*) is the projected width, *y* is distance above the substrate and *h* is the height of the colony) and the basal attachment perpendicular widths (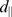 and 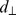) (a consistent length unit is required for all *CSF* parameters; e.g., meters). Dislodgement of an individual within the population is expected if a *DMT* generated by a wind event becomes equal to or less than its *CSF* [12]. The material density and tensile strength (*σ_t_*) of both reef substrate and *A. hyacinthus* skeleton were measured at the southeast reef at Lizard Island in an earlier study [33], which found the substrate to be approximately an order of magnitude weaker than the coral and therefore limiting whole-colony mechanical integrity under present-day environmental conditions. The expected yearly maximum water velocity (*U*) was calculated at the same reef crest site based on the reconstruction of a 37-year history of wind conditions at the study site [35]. We used this field-validated wind-fetch and wave attenuation model to calculate how changes in storm intensity (equivalent to maximum sustained wind speed) translate into maximum wave orbit water velocity at the substrate. We used the fitted curvilinear relationship between regional wind speeds *U_wind_* and colony-level water velocities at the reef crest (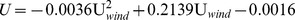, r^2^ = 0.9899) to calculate how proportional changes in storm intensity translate approximately into maximum water velocity (e.g., a 10% increase in expected storm intensity is assumed to translate in to a 10% increase in yearly maximum regional wind speed). Shifts in storm intensity in the model are based on the expected 2 to 11% increase of global intensities by 2100 [3].

**Figure 3:**
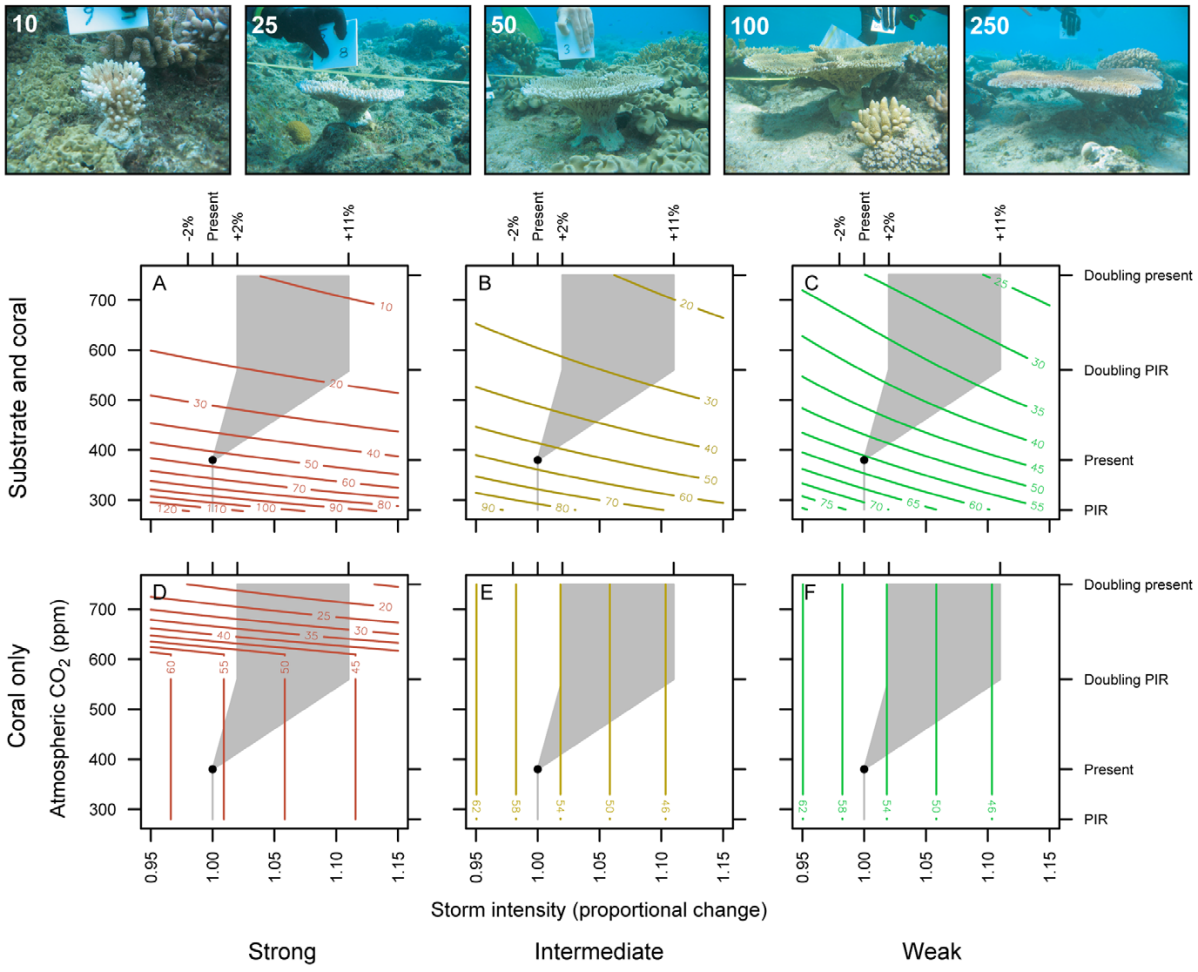
The reef coral mechanical environment. Mean expected yearly mechanical threshold (*DMT*) as a function of storm intensity and atmospheric CO_2_ scenario for reef calcification scenarios: strong (A, D), intermediate (B, E), and weak (C, F) applied to both reef substrate and coral (A–C) and coral only (D–F). The black points represent present-day estimates of mean yearly *DMT*. Shaded lines and areas represent parameters used in the IPM, including the pre-Industrial Revolution and two 2100 scenarios (doubling of Pre-Industrial Revolution [560 ppm] and doubling of present-day [750 ppm]). For reference, coral photographs illustrate *DMT* levels that would theoretically dislodge tabular colonies based on their shape.

Changes to material strength and yearly colony growth were based on proportional changes to coral calcification *G* in response to sea surface temperature (*SST*) and aragonite saturation state (Ω*_arag_*). *SST* and Ω*_arag_* estimates were used for a range of stabilized atmospheric CO_2_ levels, ranging from historical Pre-Industrial Revolution (PIR) conditions (280 ppm), through present-day (380 ppm), to two future climate change scenarios (doubling of PIR: 560 ppm; and doubling of present-day: 750 ppm) [28]. Proportional changes in calcification (relative to present-day) were estimated in three ways to capture the likely range of calcification responses of reef corals to future mean *SST* and Ω*_arag_* (Figure 2):

- The “strong” response is based on an empirically-derived equation for whole-reef aragonite precipitation (Eq. 6 in [5]), which considers both changing *SST* and Ω*_arag_*:

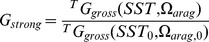
where *^T^G_gross_* is coral gross calcification and *SST* ^0^ and Ω*^arag^* ^,0^ are present-day temperature and saturation state values.

- The “intermediate” response is based on an experimentally derived relationship for reef corals in a microcosm (equation from Fig. 2 caption in [36]), which was later supplemented with other calcification studies (Eq. 1 and the first order response in Fig. 9 in [30]) and considers changes in Ω*_arag_* only. The two responses are similar and we refer to [30] from this point on.

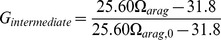

- The “weak” response is based on the linear model fitted to experimental data for a branching congener of *A. hyacinthus*, *A. intermedia*, for two SST and three Ω_arag_ experimental treatments [29], where coefficient estimates were calculated from the paper’s online supporting ANOVA table:

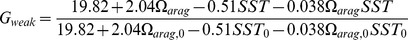



Other studies of calcification responses for the genus *Acropora* fall within this range and consider changes in Ω*_arag_* only (e.g., [37], [38]).

**Figure 4:**
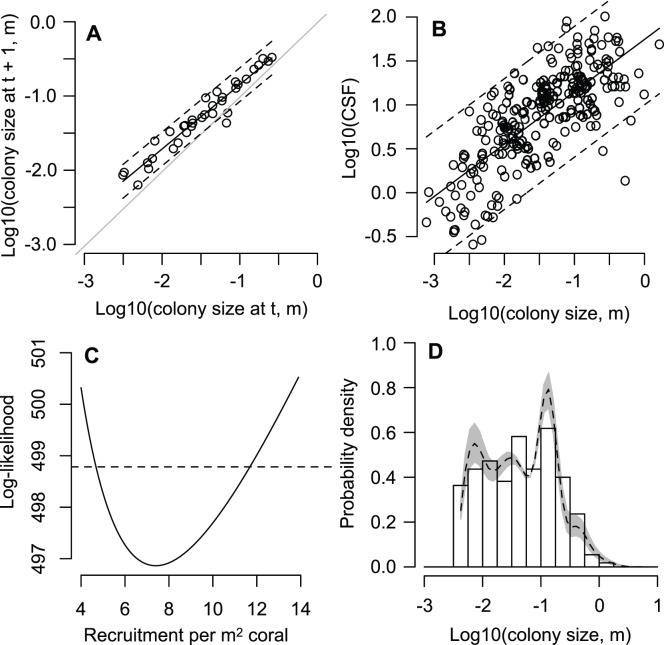
Parameterizing the population model using empirical demographic data. (A) *A. hyacinthus* colony planar area (m^2^) at year *t* +1 plotted against area at year *t* at the exposed reef crest study location. The unity line (intercept 0 and slope 1) illustrates that the majority of points fall in the region of increasing size. (B) Colony shape factor (*CSF*; dimensionless) as a function of colony planar area (m^2^) of *A. hyacinthus* colonies. Dashed lines in both panels represent 95% prediction intervals. (C) Log-likelihood profile for integral projection model recruitment parameter. The dashed line shows the log-likelihood 95% confidence bounds. (D) Colony size density distribution of *A. hyacinthus* at the study site (bars) and the best-fit model stable size distribution as a result of optimizing the recruitment parameter. Shaded area illustrates 95% log-likelihood confidence intervals.

**Figure 5:**
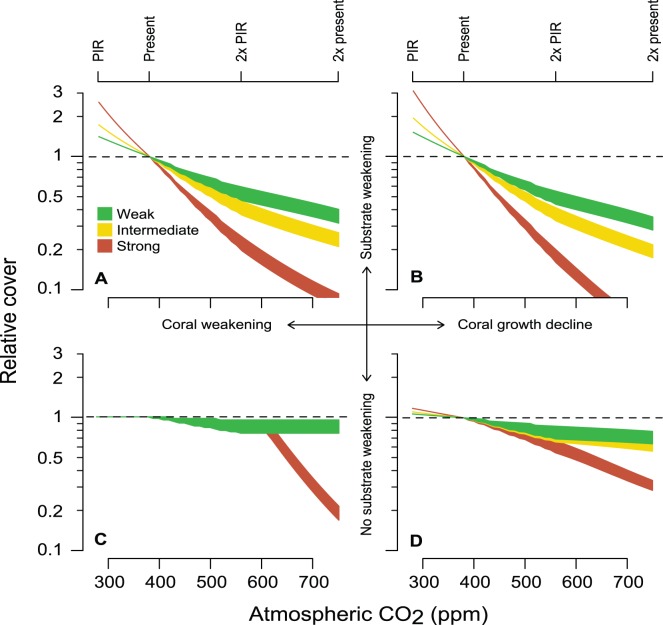
Projected coral cover under alternative future CO_2_ stabilization scenarios. pCO_2_ is assumed to affect demographic processes through different mechanisms in each panel: (A) coral and substrate weakening, (B) coral growth decline and substrate weakening, (C) coral weakening only, and (D) coral growth decline only. Curves represent the three published calcification responses to Ω_arag_ and SST: low, intermediate and high. Shaded areas capture the 2 to 11% range of predicted increases in future storm intensity [3].

The proportional change in calcification was applied to coral skeleton density or growth as per the scenarios outlined earlier (e.g., a 10% reduction in calcification relative to present-day translates into either a 10% reduction in skeletal density or a 10% reduction in added planar area per year). Changes in skeletal strength were calculated based on an empirically derived relationship between aragonite density *ρ_a_* and tensile strength (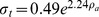) [4]. Little is known about how the material properties of reef substrate are related to ambient levels of *SST* and Ω*_arag_*. Therefore, in scenarios with reef substrate weakening, we assumed that substrate density changes similarly to skeleton density (as calcification changes), and we used the reported relationship between density and strength [4] to calculate substrate strength. Our projected estimates based on these assumptions are likely to be conservative, because substrate in the future will be comprised of coral skeleton that is presumably weaker than it is presently, and the processes cementing this skeleton are expected to diminish as aragonite saturation state declines [32]. Eq. 1 was then used to predict the mechanical environment for colonies as a function of storm intensity and calcification potential for the scenarios outlined above.

**Figure 6:**
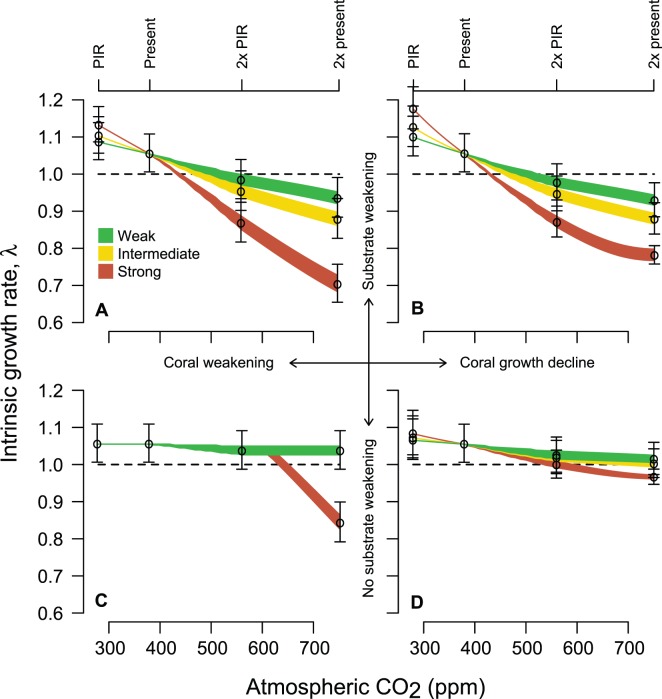
Projected long-run density-independent growth rate *λ* for the *A. hyacinthus* meta-population. Values above unity (dashed line) imply capacity for population regeneration. Points and 95% confidence intervals show uncertainty in projected *λ* due to uncertainty in the estimate of per-capita recruitment. Panels and curves correspond with the same scenarios as presented in Figure 5.

### Integral Projection Model

An integral projection model (IPM) [39] was used to translate changes in the mechanical environment into three population-level measures: cover, lifetime reproductive output, and intrinsic population growth rate. Cover is the sum of individual colony areas in a population. Lifetime reproductive output is the reproductive output of a colony or cohort integrated over the average lifespan. The intrinsic population growth rate is the per-capita propensity for population regeneration following a reduction in population size to very low levels (e.g., due to a tropical storm, bleaching event or crown of thorns outbreak). The IPM framework is well-suited to corals, because colony size is treated continuously, avoiding coarse and arbitrary size classifications, and thereby facilitating a more precise characterization of colony growth, fecundity and mechanical vulnerability, all of which are highly dependent on colony size. The parameterization of the projection model is based on a well-studied population of *A. hyacinthus* living on the physically exposed reef crest on the southeast reef at Lizard Island, Great Barrier Reef, Australia. We modeled the number of individuals in the population of size *y* at time *t* +1 given the number of individuals of size *x* at time *t* [39]:
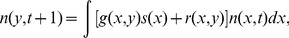
(2)which is made up of probability functions for yearly growth (*g*), survivorship (*s*), and recruitment (*r*). The growth function was estimated using permanent quadrat data at the study site (Hughes, unpublished data). The planar area of colonies of standalone (i.e., uncrowded) *A. hyacinthus* was calculated from digitized photo-quadrats from one year to the next to estimate growth rate based on the change in planar area over time (*n* = 45). Growth in standalone colonies best reflects unconstrained population growth, such as recovery following a storm disturbance while space is not limiting and competitive pressure is small. Colonies missing at *t* +1 were excluded for calculating yearly growth.

Survivorship was modeled as:
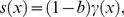
(3)where 1 - *b* is the background yearly survivorship (excluding hydrodynamic disturbance mortality), and *γ*(*x*) is the probability of dislodgment for a colony size *x* due to hydrodynamic disturbance. We develop *γ*(*x*) using previously-calculated values of *CSF* and size (planar area) for *A. hyacinthus* from the outermost, 20 m wide, section of the exposed reef crest at the study site (*n* = 220) [12]. For a given year, the expected distribution of Dislodgement Mechanical Thresholds (*DMT*s) was calculated according to Eq. 1. Therefore, the hydrodynamic disturbance survivorship function becomes:
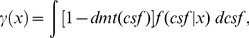
(4)where *f(csf | x)* is the probability density function of *CSF* for a colony of size *x*, and *dmt(csf)* is the Gumbel cumulative density function of minimum yearly *DMT* from 1967–2003 reported by [35].

Yearly background mortality *b* was estimated from the quadrat data as the proportion of colonies at time *t* that were dead at *t* +1. Two-thirds of the colonies that were dead at *t* +1 died during periods when Lizard Island experienced large cyclonic wave conditions (1990–1991 [Cyclone Ivor] and 1998–1999 [Cyclone Rona]). Therefore, we calculated the number of colonies that died in each year, out of the total number observed in the data (excluding the two cyclone years), and used binomial likelihood to estimated background mortality probability.

Because *A. hyacinthus* is a broadcast spawner, we first modeled the population as an open system, which assumed that recruitment is largely independent of the local population and that the majority of recruits are from habitats insensitive to the modeled climate variables. Minimum colony size was set to 10^−2.5^ m^2^ (∼7 cm diameter), which is the size of first recruitment in the IPM, corresponding to the approximate size of first reproduction for *A. hyacinthus* [19], [40]. For a given recruitment rate, we projected the population from year to year for each storm intensity and calcification rate scenario until the population stabilized (typically 5–25 years). When expressed relative to present-day cover, the differences in cover among scenarios were insensitive to initial population structure or recruitment rate. To calculate relative lifetime reproductive output, we seeded the population and projected it without recruitment until it went extinct, and then summed the total area of colonies from each year of the projection. Relative lifetime reproductive output was insensitive to the number of recruits in the projected cohort.

Because changes to storm intensity and calcification are large-scale phenomena, and *A. hyacinthus* tends to occupy the wave-exposed habitats most vulnerable to such changes, increased mortality or reduced growth of adult colonies is likely to have substantial feedback effects on reproductive output at the meta-population level, and thus recruitment is also likely to decrease over time. To assess the demographic implications of such feedback effects on recruitment, we used a closed population model, in which the supply of recruits declined in proportion to the reproductive output of the local population. The closed model assumes that we capture the global pool of adults and that variables, such as substrate strength, flow regime and mortality, are similar for all populations. We then assume that the number of colonies entering the population at *t* +1 is proportional to the total planar area of colonies in the population at *t* [19]. Specifically, recruitment was modeled as:
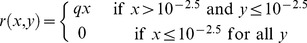
(5)where *q* is the number of colonies recruiting back to the population as 10^−2.5^ m^2^ colonies for a given area of adult colony. We estimate the recruitment parameter by varying *q* until the predicted colony size distribution (the eigenvector associated with the IPM’s leading eigenvalue [39]) best fit the empirical size probability density distribution for the study site [12]. Maximum log-likelihood was used to find the best-fit parameter *q* and the log-likelihood confidence intervals were used as estimates of uncertainty associated with the recruitment parameter. We then used the long-run density-independent population growth rate of this population, the dominant eigenvalue *λ*, to quantify the population-dynamic consequences of increases in storm intensity and declines in calcification rate, because it expresses the different demographic effects of such environmental changes in the common currency of the capacity of a coral colony to contribute to population growth [41].

## Results

### Reef Mechanical Environment

The mean expected yearly mechanical threshold (*DMT*) showed a range of potential trajectories depending on environmental change and coral response scenario (Figure 3). For scenarios in which reef substrate strength declines as a function of calcification potential–and regardless of if coral individuals invest in colony growth or skeletal density (strength)–the mean dislodgement threshold is expected to decrease by up to four-fold (a given colony is four times more likely to be hydrodynamically dislodged) by the end of the century, depending on the calcification response to changing atmospheric CO_2_ levels (Figure 3A–C). These decreases in *DMT* are driven primarily by reduction in material strength and secondarily by increases in maximum yearly water velocities (Eq. 1), and were especially pronounced for the “high” reef calcification scenario (Figure 3A). For scenarios in which reef substrate strength is unaffected by future pCO_2_ levels, and coral colonies maintain fast colony growth despite declines in skeletal density, decreases in *DMT* are driven primarily by storm intensity and change relatively little by century’s end. An exception is for the high calcification response scenario, where a point is reached at approximately 600 ppm where coral skeleton became weaker than the reef substrate, and *DMT* declines precipitously (Figure 3D). For scenarios in which substrate strength is unaffected by future pCO_2_ levels and coral colonies sacrifice colony growth in order to maintain skeletal strength, decreases in *DMT* are driven only by storm intensity (identical to that shown in Figure 3E, F).

### Integral Projection Model

Colony growth for *A. hyacinthus* colonies is well described by a power-law relationship between colony sizes in successive years (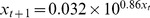, Figure 4A). The slope of the relationship is less than one (0.86), indicating that added area (as a proportion of size) declines as colonies grow. The relationship between colony mechanical vulnerability and size is also captured well by a power-law relationship (Figure 4B). We estimated the non-cyclone induced background mortality probability as 0.066 per annum (95% confidence intervals: 0.017–0.164), and assume that this level is constant from year to year. This result is consistent with estimates from other studies at Lizard Island [42].

When modeled as an open system, population projections indicate declines in *A. hyacinthus* cover of more than twofold by the end of the century in 8 of the 12 scenarios we examined (Figure 5). Projected lifetime reproductive output showed almost identical patterns relative to present-day. Variation in the magnitude of projected change among our 12 scenarios indicates that the population-level response to increasing atmospheric CO_2_ depends primarily on the sensitivity of substrate strength to reef calcification rate. Decline is rapid if the strength of the reef substrate diminishes at a rate similar to that of *in situ* measurements of community calcification rate (red curves, Figure 5A, B). Decline is intermediate if substrate strength diminishes in proportion to measurements of the calcification rate of individual coral colonies (Figure 5A, B: yellow and green curves), while decline is small if substrate strength is insensitive to Ω_arag_, and thus changes are driven primarily by storm intensity (Figure 5C, D). Regardless of the reef substrate response to acidification, population cover declines less rapidly if colonies also reduce their skeletal density to maintain growth rate (Figure 5A), except when coral skeletal strength falls below that of the reef substrate, at which point population growth rate declines precipitously (Figure 5C, red curve). Similar thresholds occur for the other intermediate and weak calcification response curves in Figure 5C, but these thresholds lie at atmospheric CO_2_ levels beyond those explored here.

When modeled as a closed system, the IPM eigenvector that best fit the empirical size structure data had a recruitment parameter *q* at time *t* +1 of 7.4 recruits per m^2^ of colony planar area at time *t* (Figure 4C). The log-likelihood profile 95% confidence interval ranged from 4.7 to 11.7 recruits per year per colony area. Given this best-fit recruitment rate, the model fitted the empirical size distribution data reasonably well (Figure 4D) and the two were statistically indistinguishable (two-sided Kolmogorov-Smirnov test: *D* = 0.2, *p* = 0.2719). Parameterizing the recruitment function with the mean and confidence intervals for *q*, the integrated projection model suggests that populations are currently able to replenish themselves (Figure 6), with a mean annual population growth rate, *λ*, of approximately 1.056 (implying a population doubling time of 12 years; 95% confidence bounds ranging from 1.007 to 1.110). By the end of this century, *λ* is predicted to decline below unity, indicating loss of the capacity for self-replenishment, in 7 of the 12 doubling Pre-Industrial Revolution and 9 of the 12 doubling present-day scenarios we examined. In contrast with predictions for coral cover, *λ* is predicted to decline marginally faster as a function of atmospheric CO_2_ level when corals maintain growth rate and sacrifice skeletal density (Figure 6A, B).

## Discussion

Our study focuses specifically on two environmental changes likely to be associated with anthropogenic effects on climate (increased storm intensity and decreasing aragonite saturation state due to the interaction between ocean acidification and increasing temperature), and it explores their demographic effects on adult coral growth and mortality from storms. The model translates these environmental changes into individual-level growth and mortality probability, thereby providing a more mechanistic basis for population-level responses than more traditional phenomenological approaches that consider only the aggregate dynamics of coral cover. Our results indicate that the environmental change scenarios we examined will impact levels of cover and population resilience of *A. hyacinthus*. The magnitude of this impact depends primarily on how SST and Ω_arag_ influences reef substrate mechanical integrity, which is currently poorly understood [32], and secondarily on the degree to which colony calcification diminishes, for which a range of scenarios exist [43]. Change in colony calcification introduces an important demographic trade-off between maintaining growth rate (greater reproductive output) and skeletal density (lower storm-induced mortality), which requires further research to understand the potential for adaptation within the physiological and energetic constraints imposed by this trade-off [44]. Finally, increases in storm intensity expected by 2100 appear to play a relatively minor role in long-term population persistence, compared to calcification responses and individual-level demographic trade-offs.

Our projections are likely to be somewhat conservative because we omit other human impacts that reduce coral reproduction and growth and/or increase mortality [45] (e.g., pollution, overfishing and other effects of rising CO_2_ and SST). For example, one of the most biologically significant effects of increasing temperatures on corals is increases in the frequency and severity of coral bleaching, which can cause mass mortality, increase susceptibility to disease, and reduce subsequent growth and reproduction [46]. The frequency and intensity of bleaching events has been increasing over the past several decades [46], and, while there is growing evidence of acclimation and adaptation to warmer temperatures [43], it is unlikely that such physiological and evolutionary changes will occur rapidly enough to avoid adverse consequences entirely [6], [29], [43]. Furthermore, our approach does not include any effect of ocean acidification on pre- and post-settlement stages [13], [14]. Finally, the combined impacts of ocean acidification, pollution, overfishing and other environmental changes are likely to change species composition and interactions [23], [26], including reductions in herbivore abundances that mediate shifts from corals to algae or other weedy species [45], [47]–[49].

By developing explicit mechanistic connections between size-dependent demographic rates and environmental feedbacks, our study makes important advances on earlier projections of the effects of climate change on coral persistence, which model the dynamics of coral cover in the aggregate [6], [7]. For instance, we find here that declining calcification potential has feedback effects on coral growth and structural integrity, which subsequently influence size structure and size-dependent mortality and fecundity. Moreover, increasingly, climate scientists have begun to rigorously incorporate uncertainty into modeling, for instance in the estimation of climate sensitivity used to project future temperature change [50]. Indeed, for projecting effects of climate change on coral reefs, many relevant biological parameters are often known with considerable uncertainty. For instance, because all relevant parameters are not known for a single focal species, parameter sets, of necessity, often include a mixture of values obtained from a range of species with sometimes very different ecologies [6], [7]. Therefore, it is important for studies of the ecological effects of climate change to take the additional step to represent the uncertainty that such errors may contribute to projections. Here, we used multiple scenarios, and estimates of measurement error for critical demographic parameters, to quantitatively project the effects of climate change on the demography of a critical engineer of habitat structure on wave-exposed reefs on many Indo-Pacific reefs, *A. hyacinthus*. Our results show that tabular corals are prone to large and rapid declines in coral cover, and to population collapse, due principally to increased vulnerability to storm-induced dislodgment as a consequence of ocean acidification and decreased lifetime reproductive output. Because the top-heavy growth forms of tabular and arborescent corals makes such species particularly susceptible to dislodgment, these effects are likely to be manifested as a shift towards lower coral cover overall, and towards coral assemblages more dominated by structurally simpler, more mechanically stable species that are less productive and offer less shelter and food for other coral reef organisms.
